# Assessing urban-rural differences in the relationship between social capital and depression among Ghanaian and South African older adults

**DOI:** 10.1371/journal.pone.0218620

**Published:** 2019-06-19

**Authors:** Dzifa Adjaye-Gbewonyo, George W. Rebok, Alden L. Gross, Joseph J. Gallo, Carol R. Underwood

**Affiliations:** 1 Department of Health, Behavior and Society, Johns Hopkins Bloomberg School of Public Health, Baltimore, Maryland, United States of America; 2 Department of Mental Health, Johns Hopkins Bloomberg School of Public Health, Baltimore, Maryland, United States of America; 3 Johns Hopkins Center on Aging and Health, Baltimore, Maryland, United States of America; 4 Department of Epidemiology, Johns Hopkins Bloomberg School of Public Health, Baltimore, Maryland, United States of America; University of Lleida, SPAIN

## Abstract

**Introduction:**

Research has demonstrated benefits of social capital on depression, but variations in this relationship by geographic characteristics such as urbanicity have rarely been investigated.

**Methods:**

Using survey data on 4,209 Ghanaian and 3,148 South African adults aged 50 and above from the World Health Organization (WHO) Study on Global AGEing and Adult Health (SAGE), exploratory and confirmatory factor analyses were conducted to extract dimensions of social capital from survey items. Structural equation models with the extracted factors were then used to estimate the associations between social capital and depression in each sample and assess differences between urban and rural settings with measurement and structural invariance tests.

**Results:**

Factor analyses suggested three dimensions of social capital representing community engagement, sociability, and trust. Urbanicity did not substantially modify the effects of social capital on depression in either setting, but urban-rural differences in the measurement and level of social capital were observed. Urban Ghanaian older adults were less socially integrated and trusting than older rural residents (standardized mean difference: -0.28, -0.24, and -0.38 for community engagement, sociability, and trust, respectively) while urban South African older adults appeared less engaged in community activities but significantly more trusting and socially active informally than older rural residents (standardized mean difference: -0.33, 0.30, and 0.17 for community engagement, sociability, and trust, respectively). Moreover, while trust was associated with a lower risk of depression in South Africa overall, sociability and trust were associated with an increased risk of depression in Ghana.

**Conclusions:**

Results indicate that the composition and average levels of social capital differ between urban and rural older adult residents in Ghana and South Africa although urban-rural differences in the strength of the association between social capital and depression were not substantial. Furthermore, the associations between social capital and depression are context-specific and are not uniformly beneficial.

## Introduction

The importance of social capital for health has been increasingly recognized and widely studied in the public health literature [1]. Although varying definitions of social capital exist, Pierre Bourdieu, who is among the notable scholars credited for the term’s popularization, described social capital essentially as the resources derived from one’s social affiliations that are obtained through expending time in social interactions. The benefits arising from this process of exchange motivate the formation and sustenance of social groups and lead to group unity [2]. In its application to public health, Robert Putnam’s conceptualization of social capital, which emphasizes civic participation and community organizations as well as networks and customs of trust and reciprocity, has been most commonly used [3]. Despite the lack of consensus in defining the concept, its basis in social interactions and relationships is a common thread, and it has often been used to encompass related constructs such as social integration, social support, participation, and social cohesion [4].

Specifically regarding mental illness, there is convincing evidence that social connections can also play a protective role. For example, individual-level measures of social capital including social participation, trust, neighborhood attachment, and sense of belonging have been shown to be negatively associated with common mental disorders [3]. Relationship quality has also shown significant negative associations with depression [5], and a recent systematic review concluded that perceived and received emotional support, perceived instrumental support and having a larger social network and a network consisting of both friends and family protect against depression [6].

The role of social connections for the mental well-being of older adults has also become a subject of study [5], with some indication that an increased likelihood of insufficient support and interaction may partly explain elevated rates of depression in this population [7]. Research likewise suggests many benefits of social support, integration, quality relationships and other aspects of social capital in terms of depressive outcomes in older adults, although the significance of findings have also varied [5].

Studies have also examined variations in the association between social relationships and depression based on different personal characteristics, and the relationship has been shown to vary by gender, age, personality traits, and even genetics [6]. However, little research has been devoted to variations in the social capital-depression association by geographic factors. For example, the question of urban-rural differences in the association between social capital and depression is largely understudied, yet urbanization is happening at a rapid rate globally and particularly in low- and middle-income countries (LMICs) [8]. Additionally, urban-rural differences have been demonstrated in the occurrence of depression itself in several cases, with results of a meta-analysis suggesting a roughly 30% increase in odds of mood disorders such as depression in urban as compared to rural settings [9]. Thus, understanding the differential effects of social capital on depression by urbanicity may be important for elucidating potential explanatory factors behind urban-rural disparities observed in depression rates, and it may have utility for informing the planning of appropriate points of interventions across these settings.

Furthermore, there is growing recognition that the effects of social relationships may differ across cultures, indicating the need for particular attention to these differences [5, 6, 10]. Yet, evidence from regions such as Africa is especially lacking. Taking these points into consideration, this study therefore examines urban-rural differences in the relationship between social capital and depression in the African context using the World Health Organization (WHO) Study on Global AGEing and Adult Health (SAGE). The WHO SAGE is a rich data source that enables us to study this research question in two middle-income African countries, Ghana and South Africa, and thus assess any potential similarities, as well as possible differences, in findings across these two locations within Africa.

We hypothesize that the association between social capital and depression is in fact modified by the type of geographic setting. More specifically, while it is expected that social capital will have protective effects on depression, we hypothesize that the strength of the association will be weaker for residents of urban areas as compared to residents of rural areas. This hypothesis is informed firstly by the fact that rural areas are generally under-resourced and suffer from inadequate health and other services compared to urban areas [11], which may make rural residents depend more heavily on their social networks for the fulfillment of support needs. As a result, they may be more vulnerable to the effects of an absence of strong social capital.

Additionally, some studies have identified effect modification of the social support-depression association by degree of urbanicity in the hypothesized direction. For example, a study in an urban and a rural area of a region in Japan identified significant associations between inadequate social support and depression only in the rural but not the urban residents after adjustment [12]; and another study in Korean older adults found a weaker association between social support deficits and depression in urban residents compared to rural residents—despite lower levels of support among urban dwellers—with a dose-response relationship according to length of urban residence and essentially no association in lifetime urban residents [13]. The authors suggested that urban individuals may place a lower value or emphasis on social relationships than rural residents, and this decreased relevance could make urban residents less affected by insufficient social support. Likewise, low levels of emotional social support had a stronger effect on psychological distress among those living in villages than in cities in former Soviet countries [14]. Thus, the hypothesized differential effects of social capital on depression may be related to the greater availability of alternative resources to compensate for social capital deficits and a lower valuation of the importance of social connectedness in urban settings.

## Data and methods

Data for this analysis were taken from the first wave of the World Health Organization (WHO) Study on Global AGEing and Adult Health (SAGE), a nationally representative population-based household survey conducted in six low- and middle-income countries [15]. Data for Ghana and South Africa were collected in 2007 and 2008 and used a stratified, multistage cluster design [16, 17]. All individuals aged 50 years and older were eligible to participate, along with one individual 18–49 years old per household [15]. The study is described in greater detail elsewhere [15]. The samples used in this analysis were restricted to 4,209 Ghanaian and 3,148 South African adults 50 years of age and older who had lived in their current location for over one year. The use of 50 years as the age cutoff for older adults was selected based on the focus of the SAGE data as well as criteria used in previous research that suggest that 50 years may be a more suitable definition for African countries based on local perceptions of aging as well as social circumstances and average life expectancies in the region [18]. The exclusion of individuals with one or fewer years of residency in their current locality was to ensure that the social capital and depression measures, which are based on the previous 12-month period, were relevant to respondents’ current location.

### Measures

#### Urbanicity

Households were classified as urban or rural based on official designations within each country. In Ghana, an urban designation is given to localities with a population of at least 5,000, and in South Africa designations incorporate land use and type of settlement [19, 20].

#### Depression

Depression in the past 12 months was defined using survey items on treatment for depression within that time period as well as the reported experience of depressive symptoms. Depression was operationalized as a binary yes/no variable based on either an affirmative response to depression treatment or satisfaction of the *International Classification of Diseases*, 10th revision (ICD-10) [21] criteria for a depressive episode based on an algorithm developed from the symptom items.

#### Social capital measures

15 items consisting of Questions 6001–6010 and 6012–6016 of the Social Cohesion section of the SAGE survey were selected as potential measures of social capital. These items assessed interpersonal interactions, participation in community and social activities, and general and group-specific trust through a combination of categorical questions. A complete list of these questions and their response formats are included Table 1.

**Table 1:** List of survey items considered for the social capital measures.

Survey Item [variable name]	Scale
*How often in the last 12 months have you…*	
Attended any public meeting in which there was discussion of local or school affairs [meet]	1 (never) to 5 (daily)
Met personally with someone you consider to be a community leader [lead]	1 (never) to 5 (daily)
Attended any group, club, society, union or organizational meeting [club]	1 (never) to 5 (daily)
Worked with other people in your neighborhood to fix or improve something [neigh]	1 (never) to 5 (daily)
Had friends over to your home [guest]	1 (never) to 5 (daily)
Been in the home of someone who lives in a different neighborhood than you do or had them in your home [visit]	1 (never) to 5 (daily)
Socialized with coworkers outside of work? [cowrk]	1 (never) to 5 (daily)
Attended religious services (not including weddings and funerals) [relig]	1 (never) to 5 (daily)
Gotten out of the house/your dwelling to attend social meetings, activities, programs or events or to visit friends or relatives [out]	1 (never) to 5 (daily)
Would you like to go out more often or are you satisfied with how much you get out of the house [adequate]	1 (more often) to 3 (not more often)
Generally speaking, would you say that most people can be trusted or that you can’t be too careful in dealing with people [trust]	Can be trusted/ can’t be too careful
Do you have someone you can trust and confide in [support]	Yes/no
First think about people in your neighborhood. Generally speaking, would you say that you can trust them [revtrstn]	1 (very great extent) to 5 (very small extent)*
Now think about people whom you work with. Generally speaking, would you say that you can trust them [revtrstw]	1 (very great extent) to 5 (very small extent)*
And how about strangers? Generally speaking, would you say that you can trust them? [revtrsts]	1 (very great extent) to 5 (very small extent)*

*coding was reversed to mirror direction of other items

### Statistical analysis

Data were analyzed separately for each country. Exploratory factor analysis (EFA) was used to identify latent factors representing dimensions of social capital underlying the social capital items. The selection of a potential range for the appropriate number of factors was guided by the number of factors with eigenvalues greater than one derived from the sample correlation matrix; assessments of scree plots of eigenvalues for the number of points above where the slope of the plot begins to level off; and parallel analysis results from principal components indicating the number of eigenvalues larger than eigenvalues produced from random data [22, 23]. Factor loadings, residual variances, and fit statistics—including the chi-square (χ^2^) test (lower test statistic and non-significant p-value indicate better fit) [24]; root mean square error of approximation (RMSEA) (<0.05 indicates very good fit, <0.08 is acceptable) [23, 25]; and standardized root mean square residual (SRMR) (<0.10 or at least 0.08 is adequate) [23, 26]—were compared from multiple potential EFA models to select the most appropriate factor solution demonstrating good fit, adequate factor loadings, minimized item residual variance, and minimal cross-loading. At least 3 items were also required per factor [26] to improve model identification.

After the factor number was determined, items with factor loadings below 0.32 or with cross-loading—i.e., factor loadings of 0.32 on more than one factor and/or a difference of less than 0.15 between the 2 highest loadings [22, 23]—were deleted one at a time and the EFA re-run until a final solution was produced. An oblique rotation (promax) was used to allow for potential correlation between the factors.

Confirmatory factor analysis (CFA) was used to verify the fit of the final factor structure obtained from the EFA. Depression was then introduced as the outcome in initial structural equation modeling (SEM) with the social capital latent factors, and secondary models adjusted for sex (male/female) and age (continuous and centered at the mean) because of known differences in the occurrence of depression by these demographic factors [27]. Adequacy of the CFA and SEM models was assessed with χ^2^ tests, RMSEA, and the comparative fit index (CFI) and Tucker-Lewis index (TLI) (> 0.9 is acceptable, ≥ 0.95 good) [23, 25, 26].

Finally, a multi-group analysis was conducted to determine whether differences existed between urban and rural residents in the SEM of the relationship between social capital latent factors and depression. Invariance, or equivalence, between the two groups for the measurement models specified in the CFA was first examined as a necessary prerequisite. This analysis assessed scalar measurement invariance, which assumes that factor loadings and item thresholds are equal across the groups and allows for comparisons of factor means [25]. A χ^2^ difference test was used to examine the null hypothesis of no significant difference between the constrained model assuming scalar invariance between urban and rural resident groups and an unconstrained model allowing factor loadings and item thresholds to vary but maintaining the same CFA structure (configural invariance) [25]. If full measurement invariance was not supported, a partially invariant measurement model was examined by allowing the most disparate factor loadings and items thresholds between urban and rural residents to vary [24, 25]. Lastly, the structural paths between the social capital factors and the depression outcome in SEM were constrained to be equal across urban and rural residents and compared to a model allowing the path estimates to vary between urban and rural residents using a χ^2^ difference test of the hypothesis of no worse performance of the constrained model compared to the unconstrained model.

Due to the categorical nature of the questions, factor analysis was based on the polychoric correlation between the items [28], and EFA, CFA, and SEM modeling employed robust weighted least squares estimation (WLSMV) to accommodate violations of normality in the categorical items and produce valid standard errors and χ^2^ test statistics [29]. WLSMV estimation with categorical factor indicators results in the use of probit regression to model relationships between indicators and factors as well as structural paths [30], which models predicted probabilities as the outcome.

Observations missing on all dependent variables (factor indicators and the depression outcome) were dropped in the analysis. In cases where only some variables were missing, EFA, CFA, and SEM were modeled with all available information assuming missing data are only dependent on observed independent variables [30]. Missingness in the two samples was minimal, generally less than 1% (n = 14) in the Ghana sample and 3.7% (n = 117) in the South Africa sample. In the South Africa data, larger numbers of the responses were missing for items related to socializing with coworkers and trust of coworkers, which appears to be a result of the high levels of unemployment in this sample.

All models adjusted the standard errors and χ^2^tests for clustering and stratification in the survey data. Due to the skewed distribution of weights with numerous outliers, model results are based on unweighted data. Analyses were conducted in STATA 13 and Mplus 7.4.

### Ethics statement

The study was reviewed by the Johns Hopkins Bloomberg School of Public Health Institutional Review Board (IRB), which determined that it does not qualify as human subjects research requiring IRB oversight because it entailed secondary analysis of existing, de-identified data publicly available from the WHO. The SAGE study itself obtained ethics approval from the WHO and respective institutions at each site, and written informed consent was obtained from the household informant and individual respondents prior to the interviews [31].

## Results

### Sample characteristics

In the Ghana sample, 47.3% of respondents were female. The age distribution of the sample was 39.1% 50–59 years, 28.1% 60–69 years, 22.9% 70–79 years, and 9.9% 80+ years. Urban residents constituted 40.9% of the sample, and 7.6% of the sample were classified as depressed in the past 12 months. Over half (55.0%) of the sample had no formal education, and 57.1% were currently married or cohabiting. 71.9% were currently employed. In South Africa, 60.1% of the sample was female. The age distribution was 43.6% 50–59 years, 32.8% 60–69 years, 17.5% 70–79 years, and 6.1% 80+ years. Roughly two-thirds (67.1%) of the sample lived in urban areas, and 4.3% had depression in the past 12 months. Additionally, 27.3% of the sample were currently employed, half (50.7%) were married/cohabiting, and a quarter (25.5%) had no formal education.

### Exploratory & confirmatory factor analyses: Ghana

In the Ghana data, 17 participants had missing or unknown values on all social capital indicators, resulting in a sample size of 4,192 for the EFA. The EFA of the Ghana data produced 5 factors with eigenvalues above one; however, a scree plot and parallel analysis suggested a 3-factor solution. After assessing EFA models from 1 to 5 factors, the 3-factor solution appeared optimal as it resulted in acceptable fit, and minimized residual variances and cross-loading while maintaining an adequate number of indictors per factor.

With the 3-factor solution established, the item on satisfaction with how often respondents go out was removed due to inadequate loadings (below 0.17) on all factors. Subsequently, the item on going out for social activities was removed due to cross-loading on factors 1 and 2. The final factor structure thus contained 13 items distributed among the 3 factors (Table 2). Based on its emphasis on involvement in neighborhood and organized group activities, the first factor was labeled “community engagement.” The second factor represented items related to informal social interaction and was named “sociability.” Finally, the third factor included the items assessing general and specific trustworthiness of others and was referred to as “trust.” Eigenvalues for the 3 factors in the final EFA model were 4.02, 2.59, and 1.57 and model fit statistics were χ^2^ = 561.10 (df: 42, p<0.001), RMSEA = 0.054, and SRMR = 0.047. Items and their factor loadings along with correlations between factors are presented in Table 2. CFA of the final factor structure also suggested that the model was appropriate based on most fit statistics (RMSEA = 0.057 (95% CI: 0.054–0.061), CFI = 0.93, and TLI = 0.92), apart from the χ^2^ (911.99 df = 62, p<0.001). All model parameter estimates were significant except for the correlation between factor 2 (sociability) and factor 3 (trust), which was only borderline significant. Standardized CFA results are also displayed in Table 2.

**Table 2:** Factor loadings of social capital dimensions by country.

	Ghana						South Africa					
	Final EFA			CFA			Final EFA			CFA		
Item	Factor 1 (CE)	Factor 2 (S)	Factor 3 (T)	Factor 1 (CE)	Factor 2 (S)	Factor 3 (T)	Factor 1 (CE)	Factor 2 (S)	Factor 3 (T)	Factor 1 (CE)	Factor 2 (S)	Factor 3 (T)
*Attended public meeting*	0.810	-0.183	-0.024	0.674			0.869	-0.144	-0.003	0.812		
*Met with leader*	0.656	0.090	0.051	0.725			0.863	-0.131	-0.002	0.811		
*Attended club meeting*	0.677	0.103	-0.015	0.724			0.745	0.034	-0.003	0.756		
*Neighborhood improvement*	0.847	-0.012	0.007	0.838			0.668	0.103	0.010	0.712		
*Had friends over*	-0.019	0.831	0.046		0.863		-0.160	0.936	-0.033		0.797	
*Visited with someone from other neighborhood*	-0.030	0.920	-0.045		0.850		-0.023	0.786	-0.018		0.818	
*Socialized with coworker*	0.466	0.276	-0.141	0.567			0.391	0.242	-0.054	0.515		
*Religious attendance*	0.164	0.320	0.019		0.455		-	-	-	-	-	-
*Left house for outing*	-	-	-	-	-	-	0.164	0.456	0.101		0.585	
*Most people can be trusted*	0.051	0.135	0.630			0.675	-	-	-	-	-	-
*Have confidant*	-0.068	0.107	0.535			0.517	-	-	-	-	-	-
*Can trust neighbors*	-0.157	0.128	0.900			0.824	0.017	-0.057	0.805			0.786
*Can trust coworkers*	0.068	-0.164	0.884			0.898	-0.012	-0.013	0.940			0.941
*Can trust strangers*	0.255	-0.195	0.547			0.629	-0.018	0.060	0.622			0.640
**Factor Correlations**												
CE with S	0.393			0.464			0.349			0.328		
CE with T	0.263			0.268			0.068			0.045		
S with T	0.065			0.059			0.251			0.243		

Note: reported CFA parameter estimates are standardized with factor variances fixed to 1 for ease of comparison to EFA results

CE: Community Engagement; S: Sociability; T: Trust

### Exploratory & confirmatory factor analyses: South Africa

In the South Africa data, 121 participants had missing or unusable responses to all potential social capital indicators and 1 was missing cluster and stratification information, resulting in a sample size of 3,026 for the EFA. EFA of the sample correlation matrix resulted in 6 eigenvalues exceeding one while the scree plot and parallel analysis identified a 4-factor solution. However, EFA models were run comparing 1 to 6 factor solutions, and the 3-factor solution demonstrated improved fit statistics and residual variances while minimizing cross-loading and over-factoring and maintaining at least 3 items per factor compared to other models. As a result, a 3-factor solution was also selected for the South Africa data.

Similar to the Ghana results, the 3 factors represented community engagement, sociability, and trust (Table 2). However, there were slight differences in the corresponding items. As in Ghana, the item on adequacy of outings had very low loadings on all factors and was removed. Though the items on general trust and having a confidant also loaded highest on the trust factor, their loadings were in the 0.2 range and did not meet the minimum inclusion criteria, so they were likewise removed. Similarly, the item on attending religious activities loaded highest on sociability, as in Ghana, but did not meet the cutoff and showed some degree of cross-loading and was subsequently dropped. Rather, the item on going out for social activities loaded more strongly in the South Africa data than Ghana and was retained in the sociability factor. Thus, the final 3-factor solution contained 11 items. Final eigenvalues were 3.43, 2.33, and 1.57 and fit statistics for the EFA model were χ^2^ = 411.12 (df = 25, p<0.001), RMSEA = 0.071, and SRMR = 0.043. CFA verifying this solution demonstrated good model fit, with χ^2^ of 550.97 (df = 41, p<0.001), RMSEA = 0.064 (95% CI: 0.059–0.069), CFI = 0.95, and TLI = 0.94. The trust and community engagement factors were not significantly correlated though each was correlated with sociability. EFA and CFA factor loadings and factor correlations are presented in Table 2.

### Structural equation modeling: Ghana

**Fig 1:**
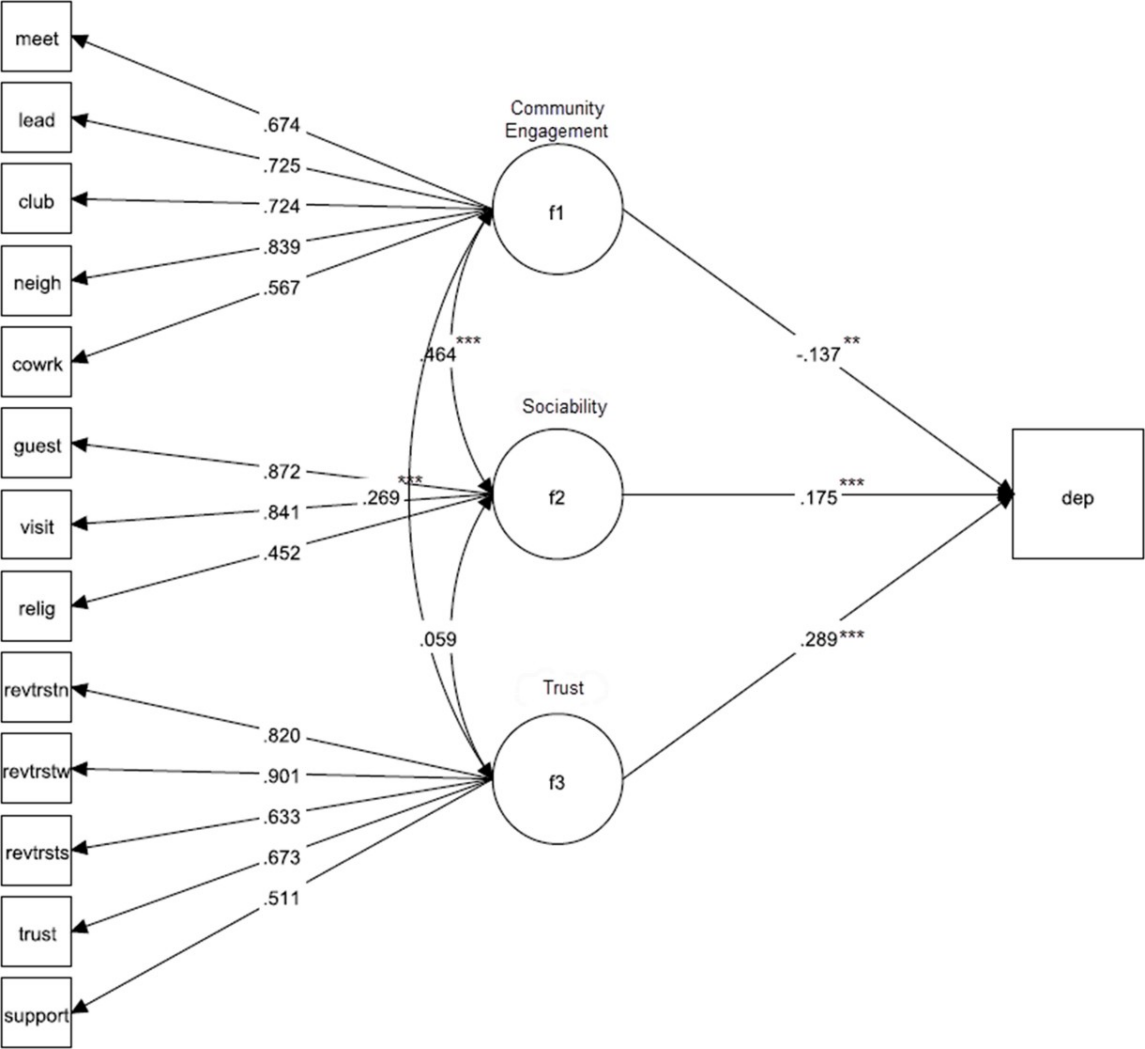
Structural equation model of the relationship between social capital and depression in the Ghana sample. *p<0.05; **p<0.01; ***p<0.001 (all factor loadings are significant at p<0.001).

In Ghana, 14 participants were missing data on all variables, resulting in a sample size of 4,195 for the SEM. Fit statistics for the model were acceptable (χ^2^ = 1058.50 (df = 72, p<0.001), RMSEA = 0.057 (95% CI: 0.054–0.060), CFI = 0.92, TLI = 0.90). Model results indicated that increases in the community engagement factor score significantly decreased the predicted probability of depression (standardized estimate: -0.14, p = 0.005) while increases in the sociability and trust factors significantly increased the predicted probability of depression (standardized estimates: 0.18 and 0.29, respectively, p<0.001). A diagrams of the general SEM for Ghana with the final CFA and depression as the outcome is illustrated in Fig 1 with its standardized parameter estimates.

Age and sex were then added to the model to adjust for their effects on depression and the latent social capital variables. The addition of age and sex to the model attenuated the relationship between community engagement and depression such that it was no longer significant (p = 0.31); however, sociability and trust remained positively and significantly linked to depression (p<0.001). Additionally, female sex had a significant direct effect increasing the predicted probability of depression, and female sex led to lower levels of all three social capital dimensions, though this was only significant at the 10% alpha level for sociability (p = 0.093). Increased age also significantly increased the probability of depression directly and was linked to significantly lower levels of community engagement and sociability (p<0.001) but did not have a significant effect on trust (p = 0.31). Model fit statistics were reasonable, with a χ^2^ of 1216.74 (df = 92, p<0.001), RMSEA = 0.054 (95% CI: 0.051–0.057), CFI = 0.91, and TLI = 0.89.

### Structural equation modeling: South Africa

**Fig 2:**
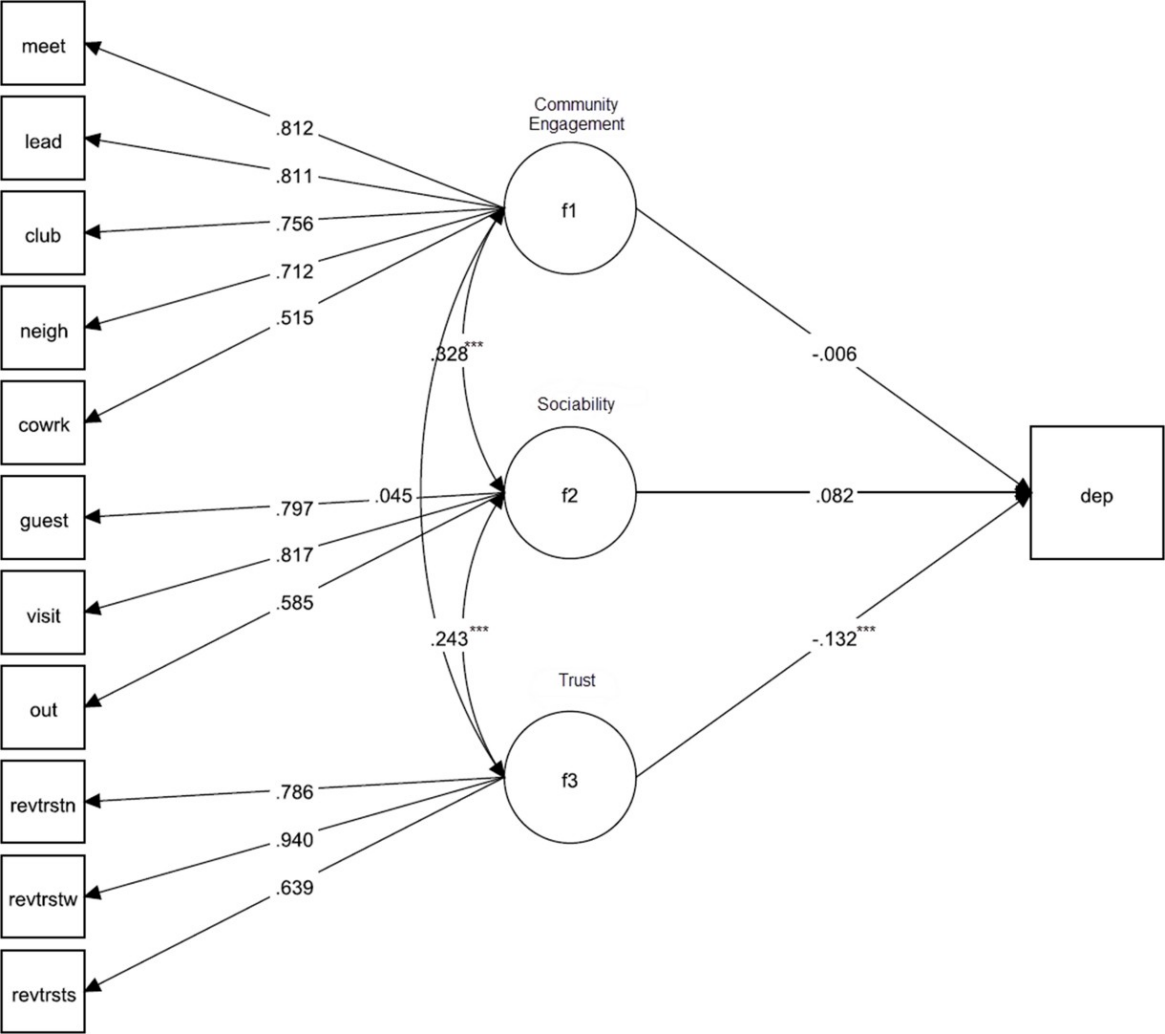
Structural equation model of the relationship between social capital and depression in the South Africa sample. *p<0.05; **p<0.01; ***p<0.001 (all factor loadings are significant at p<0.001).

In the South Africa sample, 103 participants were missing data on all variables and 1 lacked cluster and strata information, leading to a sample size of 3,044 for the structural equation models. Model fit was also acceptable, χ^2^ = 573.67 (df: 49, p<0.001), RMSEA = 0.059 (95% CI: 0.055–0.064), CFI = 0.95, TLI = 0.94. Results indicated that community engagement was not significantly associated with depression (p = 0.90), though the estimate was slightly negative. Sociability was also only significant at the 10% alpha level but as a positive predictor of depression (standardized estimate: 0.082, p = 0.093); however, increased trust significantly reduced the predicted probability of depression (standardized estimate: -0.132, p<0.001). The SEM diagram for South Africa is illustrated in Fig 2.

The addition of age and sex did not change the nature of the relationships between social capital and depression. Increasing age, however, directly decreased the predicted probability of depression (p<0.001) while sex had no significant direct effect on depression (p = 0.26). Female sex and increasing age also led to significantly lower levels of community engagement (p<0.001) while age significantly decreased sociability (p<0.001) but sex had no effect (p = 0.23). Trust was not significantly affected by sex or age (p = 0.49 and p = 0.31, respectively). Model fit indices had the following values: χ^2^ = 645.28 (df: 65, p<0.001); RMSEA = 0.054 (95% CI: 0.050–0.058); CFI = 0.95, and TLI = 0.93. Parameter estimates for adjusted SEM models for both countries are presented in Table 3.

**Table 3:** Standardized adjusted structural equation model results^a^.

	Ghana		South Africa	
	Estimate	Standard Error	Estimate	Standard Error
**Predictors**				
Community Engagement→Depression	-0.052	0.052	-0.030	0.048
Sociability→Depression	0.151***	0.037	0.072	0.049
Trust→Depression	0.276***	0.039	-0.132**	0.042
Sex→Depression	0.259***	0.063	0.099	0.087
Age→Depression	0.013***	0.003	-0.020***	0.005
Sex→Community Engagement	-0.425***	0.033	-0.212***	0.045
Age→Community Engagement	-0.024***	0.002	-0.017***	0.002
Sex→Sociability	-0.066	0.039	0.052	0.043
Age→Sociability	-0.009***	0.002	-0.013***	0.002
Sex→Trust	-0.155***	0.036	-0.027	0.038
Age→Trust	0.002	0.002	0.002	0.002
**Factor Residual Covariances**				
Community Engagement WITH Sociability	0.454***	0.024	0.321***	0.030
Community Engagement WITH Trust	0.273***	0.029	0.054	0.039
Sociability WITH Trust	0.059	0.031	0.247***	0.029

*p<0.05

**p<0.01

***p<0.001

^a^Standardization uses only the variances of the latent factors and not the outcome or covariates because of their binary form, which would not result in meaningful interpretation if standardized.

### Analysis of urban-rural differences: Ghana

The initial test to establish configural invariance of the measurement model between urban and rural Ghanaian residents—which assumes the latent factors are each represented by the same items but allows factor loadings and thresholds for item categories to vary between urban and rural groups—suggested acceptable fit (Table 4). The scalar model testing strong measurement invariance with factor loadings and thresholds constrained to be equal between the urban and rural groups was not supported as the χ^2^ difference test indicated that constraining loadings to be the same for urban and rural residents was significantly worse than allowing them to vary (χ^2^ = 181.67, df = 40, p<0.001). Subsequently, partial measurement invariance models were run successively freeing indicators with large differences in unstandardized loadings or large modification indices. After allowing 5 of the 13 item loadings and their thresholds to vary (attending club meetings, meeting with a community leader, attending religious services, trusting coworkers, and having a confidant) the χ^2^ difference test reached non-significance (25.84, df = 23, p = 0.31). Additional model fit parameters are in Table 5.

**Table 4:** Results of structural equation models comparing urban to rural residents: Ghana^a^.

	Unadjusted				Adjusted			
	Rural		Urban		Rural		Urban	
	Estimate	SE	Estimate	SE	Estimate	SE	Estimate	SE
**Factor Loadings**								
F1 (Community Engagement) BY								
Neighborhood improvement	1.253	0.039	1.253	0.039	1.282	0.042	1.282	0.042
Attended public meeting	1.000	0.000	1.000	0.000	1.000	0.000	1.000	0.000
*Met with leader*	*1*.*023*	*0*.*036*	*1*.*166*	*0*.*057*	*1*.*025*	*0*.*038*	*1*.*167*	*0*.*060*
*Attended club meeting*	*1*.*179*	*0*.*038*	*1*.*048*	*0*.*060*	*1*.*183*	*0*.*041*	*1*.*050*	*0*.*062*
Socialized with coworker	0.840	0.037	0.840	0.037	0.824	0.038	0.824	0.038
F2 (Sociability) BY								
Had friends over	1.000	0.000	1.000	0.000	1.000	0.000	1.000	0.000
Visited with someone from other neighborhood	0.950	0.039	0.950	0.039	0.940	0.041	0.940	0.041
*Religious attendance*	*0*.*549*	*0*.*038*	*0*.*541*	*0*.*049*	*0*.*546*	*0*.*039*	*0*.*521*	*0*.*050*
F3 (Trust) BY								
*Can trust coworkers*	*1*.*555*	*0*.*055*	*1*.*153*	*0*.*059*	*1*.*555*	*0*.*055*	*1*.*160*	*0*.*059*
Can trust neighbors	1.319	0.038	1.319	0.038	1.323	0.038	1.323	0.038
Can trust strangers	1.000	0.000	1.000	0.000	1.000	0.000	1.000	0.000
Most people can be trusted	1.084	0.057	1.084	0.057	1.079	0.057	1.079	0.057
*Have confidant*	0.912	0.065	0.629	0.060	0.913	0.065	0.630	0.061
**Factor Variances/Covariances^b^**								
Community Engagement WITH Sociability	0.240***	0.019	0.303***	0.030	0.229***	0.020	0.293***	0.031
Community Engagement WITH Trust	0.094***	0.015	0.133***	0.025	0.088***	0.015	0.138***	0.024
Sociability WITH Trust	-0.004	0.022	0.069*	0.030	-0.005	0.022	0.071*	0.030
Community Engagement Variance	0.443***	0.025	0.444***	0.045	0.419***	0.025	0.418***	0.043
Sociability Variance	0.741***	0.033	0.724***	0.085	0.749***	0.034	0.741***	0.093
Trust Variance	0.352***	0.022	0.579***	0.062	0.351***	0.022	0.568***	0.061
**Predictors**								
Community Engagement →Depression	-0.114	0.091	-0.296**	0.105	0.007	0.093	-0.151	0.109
Sociability → Depression	0.203***	0.055	0.179*	0.084	0.170**	0.050	0.154	0.079
Trust → Depression	0.436***	0.084	0.443***	0.086	0.433***	0.081	0.406***	0.085
Sex→ Depression					0.267**	0.082	0.241*	0.098
Age→ Depression					0.012*	0.005	0.016***	0.004
Sex→ Community Engagement					-0.261***	0.033	-0.311***	0.039
Age→ Community Engagement					-0.017***	0.002	-0.018***	0.002
Sex→ Sociability					0.003	0.046	-0.119*	0.051
Age→ Sociability					-0.009***	0.002	-0.006**	0.002
Sex→ Trust					-0.092**	0.029	-0.075	0.042
Age→Trust					-0.001	0.001	0.004*	0.002
**Factor Means^c^**								
Community Engagement	0.000	0.000	-0.187***	0.044	0.000	0.000	-0.152**	0.052
Sociability	0.000	0.000	-0.203**	0.065	0.000	0.000	-0.165*	0.076
Trust	0.000	0.000	-0.291***	0.049	0.000	0.000	-0.304***	0.055

SE: Standard Error

*p<0.05

**p<0.01

***p<0.001 (all factor loadings are significant at p<0.001)

Factor loadings freed between urban and rural groups to establish partial measurement invariance are in italics

^a^ For purposes of comparison between the two groups, results presented are unstandardized and for the models without equality constraints on the social capital-depression relationships (Models 4 & 6 in Table 5). Factor loadings fixed to 1 represent the reference variable used to scale the factor.

^b^ In the adjusted model with age and sex predicting the factors, these values represent residual variances or covariances/correlations in residual errors

^c^ In the adjusted model, these values represent intercepts for the factors. The rural group served as the reference for comparison of factor means

**Table 5:** Model fit statistics for invariance testing^a^.

Model	χ^2^	df	RMSEA (95% CI)	CFI	TLI	χ ^2^ Difference Test
**Ghana**						
1: Measurement non-invariance (configural model/unconstrained loadings & thresholds)	1032.932***	124	0.059 (0.056–0.062)	0.928	0.910	
2: Measurement invariance (scalar model/constrained loadings & thresholds)	1116.849***	164	0.053 (0.050–0.056)	0.925	0.928	2 vs 1: 181.667 (df: 40, p<0.001)
3: Partial measurement invariance (selected loadings & thresholds unconstrained)	1019.982***	147	0.053 (0.050–0.056)	0.931	0.927	3 vs. 1: 25.844 (df: 23, p = 0.3083)
4: Structural non-invariance (unconstrained structural paths between factors & depression)	1161.141***	167	0.053 (0.050–0.056)	0.920	0.913	
5: Structural invariance (constrained structural paths between factors & depression)	1084.676***	170	0.051 (0.048–0.054)	0.927	0.922	5 vs. 4: 3.605 (df: 3, p = 0.3073)
6: Structural non-invariance w/ covariates	1329.883***	207	0.051 (0.048–0.053)	0.909	0.895	
7: Structural invariance w/ covariates	1252.485***	210	0.049 (0.046–0.051)	0.915	0.904	7 vs. 6: 2.922 (df: 3, p = 0.4039)
**South Africa**						
1: Measurement non-invariance (configural model/unconstrained loadings & thresholds)	601.441***	82	0.065 (0.060–0.070)	0.955	0.940	
2: Measurement invariance (scalar model/constrained loadings & thresholds)	602.657***	120	0.052 (0.048–0.056)	0.958	0.962	2 vs 1: 83.816 (df: 38, p<0.001)
3: Partial measurement invariance (selected loadings & thresholds unconstrained)	575.585***	112	0.052 (0.048–0.057)	0.960	0.961	3 vs. 1: 34.993 (df: 30, p = 0.2429)
4: Structural non-invariance (unconstrained structural paths between factors & depression)	615.913***	128	0.050 (0.046–0.054)	0.958	0.957	
5: Structural invariance (constrained structural paths between factors & depression)	585.680***	131	0.048 (0.044–0.052)	0.961	0.961	5 vs. 4: 0.972 (df: 3, p = 0.8080)
6: Structural non-invariance w/ covariates	660.830***	160	0.045 (0.042–0.049)	0.956	0.951	
7: Structural invariance w/ covariates	640.256***	163	0.044 (0.040–0.047)	0.958	0.954	7 vs. 6: 1.107 (df: 3, p = 0.7754)

*p<0.05

**p<0.01

***p<0.001

^a^Chi-square difference testing for WLSMV estimation is not calculated from chi-square values in the same manner as standard difference testing.[30] Additionally, the behavior of other fit statistics for WLSMV estimation with categorical indicators can be irregular, limiting direct comparison of their magnitudes. For this reason, constrained model fit statistics may not always appear to have worse values than their unconstrained counterparts.

The partial measurement invariance model was used in structural models to assess whether the paths between the three social capital latent variables and depression differed between urban and rural groups, suggesting effect modification by urbanicity. Results of these models indicated that constraining the structural paths to be equal was not appreciably worse than allowing them to vary between the two groups, implying that structural invariance could be assumed (χ^2^ difference test: 3.61, df = 3, p = 0.31). The social capital-depression paths for both the constrained and unconstrained models mirrored the overall SEM results reported above. However, the stratified analysis showed that the correlation between trust and sociability, which was smallest in both groups, was not significant in the rural group (p = 0.85) but reached significance in the urban group (p = 0.02). Additionally, based on the unconstrained model that allowed for group differences in the structural paths, the path for factor 1 (community engagement) to depression did not reach significance in the rural group (p = 0.30), so the overall significance in this association was primarily driven by the urban group (Table 4).

In the age- and sex- adjusted version of the test for structural invariance, there was likewise no significant difference in models allowing social capital-depression relationships between urban and rural groups to be the same rather than different (χ^2^difference test: 2.92, df = 3, p = 0.40). As in the SEM results reported previously, in both unconstrained and constrained versions of the model community engagement was no longer significantly associated with depression for either urban or rural residents. However, based on the unconstrained model, sociability and trust were again significant positive predictors of depression in the rural group (p = 0.001 and p<0.001, respectively) while sociability only trended towards significance in the urban group (p = 0.052). An additional difference revealed in the stratified output was that female sex significantly decreased trust in the rural sample (p = 0.002) but was only borderline significant in the urban group (p = 0.072). Additionally, female sex was a significant predictor of lower sociability in the urban group (p = 0.019) but had no effect on sociability in the rural group (p = 0.94), and age significantly increased trust in the urban group (p = 0.035) but was not significant in the rural group (p = 0.32).

All CFA and SEM models also indicated a significantly lower mean for the three social capital latent factors among urban residents compared to rural residents (Table 4). Standardized versions of these differences in means for the unadjusted and adjusted models, respectively, were -0.28 and -0.22 for community engagement, -0.24 and -0.19 for sociability, and -0.38 and -0.40 for trust. These approximate Cohen’s *d* effect sizes and their values represent small to moderate average decreases in levels of social capital in urban compared to rural residents. The translation into effect size for trust, however, is likely a conservative estimate since it is standardized by only the urban group’s variance, which was substantially larger than the rural variance for that factor.

### Analysis of urban-rural differences: South Africa

In South Africa, the configural model allowing factor loadings and thresholds to vary between urban and rural groups indicated good model fit (Table 5). The scalar model constraining factor loadings and thresholds to be equal between the urban and rural groups did not support measurement invariance, with a significant value for the χ^2^ difference test (83.82, df = 38, p<0.001). Models were then run to test partial measurement invariance, and after allowing 2 of the 11 item loadings (attending public meetings and meeting with a community leader) and their thresholds to vary, the χ^2^ difference test lost significance (34.99, df = 30, p = 0.24) and the measurement model achieved partial invariance.

Using the partially invariant measurement model to assess urban-rural differences in the relationships between the three social capital domains and depression, the SEM allowing the paths to differ between groups resulted in acceptable fit (Table 5). In the rural group, associations between social capital factors and depression did not reach statistical significance although trust was significant at the 10% level (p = 0.072) and had a negative parameter estimate. Community engagement also had a negative estimate though its p-value suggested essentially no effect (p = 0.883) while sociability was in the positive direction (p = 0.139). In the urban group, community engagement and sociability had positive parameter estimates, although neither was significant (p = 0.632 and 0.479, respectively); and trust significantly decreased the predicted probability of depression (p = 0.009). There was also a slight difference in factor correlations as community engagement and trust were not associated in the rural group (p = 0.987) and had only a borderline significant correlation (p = 0.090) in the urban group. The constrained version of the model also demonstrated good fit (Table 4), and the χ^2^ difference test between the two models was not significant (0.97, df = 3, p = 0.808), indicating that there was no substantial difference in the association between social capital and depression between urban and rural residents.

After accounting for age and sex, in the rural group the factors maintained the same pattern of relationships with depression as before, with trust being nearly significant (p = 0.052); but neither sex nor age was a significant predictor of depression directly (p = 0.46 and p = 0.27, respectively). Age also maintained the same relationship with social capital for rural residents as in the overall model but sex was not significantly related to any of the social capital dimensions. Among urban residents, the relationships between social capital dimensions and depression were likewise similar. However, unlike in the rural case, age did reach significance as a direct predictor of depression (p<0.001), reducing the probability of the condition as it increased. Additionally, results differed from the rural sample in that female sex significantly reduced community engagement (p<0.001). When the structural paths between the social capital factors and depression were fixed to be equal in both groups, the outcome mirrored that in the unadjusted model and was not significantly worse than the unconstrained model (χ^2^ difference test = 1.11, df = 3, p = 0.78). Fit statistics for all models used to test invariance are contained in Table 4 and parameter estimates for the structural models comparing urban and rural South Africans are presented in Table 6.

**Table 6:** Results of structural equation models comparing urban to rural residents: South Africa^a^.

	Unadjusted^b^				Adjusted^b^			
	Rural		Urban		Rural		Urban	
	Estimate	SE	Estimate	SE	Estimate	SE	Estimate	SE
**Factor Loadings**								
F1 (Community Engagement) BY								
*Attended public meeting*	*1*.*248*	*0*.*053*	*0*.*926*	*0*.*055*	*1*.*302*	*0*.*055*	*0*.*975*	*0*.*059*
*Met with leader*	*1*.*162*	*0*.*044*	*0*.*960*	*0*.*057*	*1*.*221*	*0*.*051*	*1*.*012*	*0*.*062*
Attended club meeting	1.000	0.000	1.000	0.000	1.059	0.045	1.059	0.045
Neighborhood improvement	0.957	0.039	0.957	0.039	1.000	0.000	1.000	0.000
Socialized with coworker	0.720	0.051	0.720	0.051	0.736	0.057	0.736	0.057
F2 (Sociability) BY								
Visited with someone from other neighborhood	1.000	0.000	1.000	0.000	1.208	0.063	1.208	0.063
Had friends over	0.992	0.048	0.992	0.048	1.191	0.062	1.191	0.062
Left house for outing	0.832	0.041	0.832	0.041	1.000	0.000	1.000	0.000
F3 (Trust) BY								
Can trust coworkers	1.567	0.068	1.567	0.068	1.536	0.066	1.536	0.066
Can trust neighbors	1.160	0.040	1.160	0.040	1.169	0.039	1.169	0.039
Can trust strangers	1.000	0.000	1.000	0.000	1.000	0.000	1.000	0.000
**Factor Variances/Covariances^c^**								
Community Engagement WITH Sociability	0.239***	0.021	0.226***	0.035	0.188***	0.018	0.174***	0.027
Community Engagement WITH Trust	0.000	0.020	0.043	0.026	0.003	0.019	0.046	0.025
Sociability WITH Trust	0.071**	0.022	0.129***	0.019	0.062**	0.018	0.108***	0.016
Community Engagement Variance	0.475***	0.037	0.708***	0.085	0.430***	0.034	0.619***	0.077
Sociability Variance	0.605***	0.034	0.634***	0.075	0.412***	0.040	0.433***	0.050
Trust Variance	0.373***	0.028	0.337***	0.032	0.378***	0.028	0.341***	0.034
**Predictors**								
Community Engagement → Depression	-0.022	0.148	0.029	0.061	-0.029	0.156	-0.028	0.063
Sociability → Depression	0.163	0.110	0.053	0.075	0.191	0.133	0.063	0.092
Trust → Depression	-0.191	0.106	-0.232**	0.089	-0.208	0.107	-0.229**	0.088
Sex→ Depression					0.123	0.164	0.084	0.108
Age→ Depression					-0.010	0.009	-0.024***	0.006
Sex→ Community Engagement					-0.055	0.041	-0.219***	0.054
Age→ Community Engagement					-0.007**	0.002	-0.021***	0.003
Sex→ Sociability					0.004	0.052	0.041	0.034
Age→ Sociability					-0.009***	0.002	-0.007***	0.002
Sex→ Trust					0.017	0.040	-0.035	0.027
Age→ Trust					0.003	0.002	0.001	0.002
**Factor Means^d^**								
Community Engagement	0.000	0.000	-0.275**	0.085	0.000	0.000	-0.162	0.094
Sociability	0.000	0.000	0.239***	0.064	0.000	0.000	0.180**	0.055
Trust	0.000	0.000	0.098*	0.045	0.000	0.000	0.123*	0.052

SE: Standard Error

*p<0.05

**p<0.01

***p<0.001 (all factor loadings are significant at p<0.001)

Factor loadings freed between urban and rural groups to establish partial measurement invariance are in italics

^a^ For purposes of comparison between the two groups, results presented are unstandardized and for the models without equality constraints on the social capital-depression relationships (Models 4 & 6 in Table 5). Factor loadings fixed to 1 represent the reference variable used to scale the factor.

^b^ Different reference variables were used to scale the factors in the adjusted model than in the unadjusted model due to failure of the model to converge

^c^ In the adjusted model with age and sex predicting the factors, these values represent residual variances or covariances/correlations in residual errors

^d^In the adjusted model, these values represent intercepts for the factors. The rural group served as the reference for comparison of factor means

All models for South Africa suggested that the mean for the latent community engagement factor was significantly lower in urban residents as compared to rural residents while urban residents scored significantly higher on average for the sociability and trust latent factors than their rural counterparts. However, after accounting for age and sex, the mean level of community engagement for an average aged urban man was only borderline significant (p = 0.085). Respectively, the Cohen’s *d* equivalent of the unadjusted and adjusted mean differences between urban and rural residents in the dimensions of social capital were -0.33 and -0.20 for community engagement, 0.30 and 0.27 for sociability, and 0.17 and 0.21 for trust. Thus, mean differences in dimensions of social capital between the two groups are not large, but the community engagement estimate is likely conservative given the much larger variance in the urban compared to the rural sample for that factor.

## Discussion

### Dimensions of social capital

The results of the exploratory factor analysis revealed that the selected social capital items were distributed into three dimensions in both countries, namely community engagement, sociability, and trust. Although there were some differences in the composition of these three latent constructs between countries, the included items had their largest loadings on the same factors across the two countries regardless of whether the item met the criteria for retention. This provides evidence in support of the validity of the identified dimensions and a common core structure between the two countries. The dimensions extracted from this analysis also mirror the groupings of social action, sociability, and trust and solidarity, respectively, used by Ramlagan et al. [32] for the selected SAGE survey items, though the categories in their study were not empirically derived. Interestingly, in both countries, socializing with coworkers outside of work was more strongly linked to factor 1, community engagement, than factor 2, sociability, which differs from the classification assumed by Ramlagan and colleagues [32] and was contrary to expectations.

### Social capital and depression

Although it was hypothesized that the social capital factors would be significantly and negatively associated with depression, findings suggesting sociability and trust were actually positively associated with depression in the Ghana sample contradicted this. An analysis of Ghana SAGE data by Ayernor [33] similarly demonstrated that those who had daily or weekly interaction with social ties had significantly greater odds of the depressive symptoms of sadness and/or loss of interest than those with less frequent interaction.

Likewise, although the relationship between trust and depression fit expectations in the South Africa sample, neither community engagement nor sociability were significant and sociability was in the positive direction. In the Ramlagan et al. [32] analysis of South Africa SAGE data, of the 3 social capital components corresponding to the factors in the present analysis, only the trust and solidarity component was significantly negatively associated with depressive affect in the past 30 days. And an analysis by Peltzer and Phaswana-Mafuya [34] that created an index from nine items corresponding to those in factors 1 and 2 also found no significant difference in index scores based on depression status. Thus, the findings of the current study are corroborated by other analyses of SAGE data and do not appear to be spurious.

One potential reason for some of the unexpected positive and non-significant relationships with depression in the present analysis could be related to the data’s cross-sectional nature. Thus, even though models attempted to estimate directed relationships, temporality cannot be verified, and they may be capturing other potentially reverse-causal relationships. However, most research on social relationships and depression has also been cross-sectional [6]; and in comparing cross-sectional and longitudinal studies of older adults on the topic, Schwarzbach et al. [5] found little difference in findings for most facets of social relationships except for measures of social integration, which were more consistent in longitudinal studies but often failed to demonstrate an effect cross-sectionally. They attributed this to a potential stronger preventive role of social activities but little effect among the already depressed.

As the review indicates, the beneficial role of social capital in relation to depression is not always supported empirically and, in particular, there may be a strong dependence on the type of social capital assessed. In general, the strongest evidence on social relationships and depression seems to come from measures of social support, and more specifically, perceived support as compared to received support [6]. Yet, only 1 item in the SAGE survey assessed what could be viewed as perceived emotional support, which may also play a role in the observed results. Likewise, the review by Schwarzbach et al. [5] concluded that qualitative aspects of social relations were more consistently linked to protection from depression than quantitative aspects of social networks, and the De Silva et al. [3] review also found stronger evidence for cognitive forms of social capital (i.e., trust, sense of belonging) while evidence for structural social capital (i.e., social participation and networks) was less decisive. An analysis of national data from South Africa also found a negative association between social trust and depression but no effect of civic participation [35]. These findings lend support to the results of the present study, particularly for South Africa in which trust had the strongest and only significant effect estimate overall. And although in Ghana the direction of effect was reversed, trust was also most strongly linked to depression.

Another consideration in terms of unexpected findings in the relationships between social capital and depression could relate to the measure of depression itself. Because the main depressive symptom questions in the SAGE survey are based on a structured diagnostic interview and contain skip patterns designed to align with recognized clinical criteria for depression that require prerequisite symptoms, it limited the ability to evaluate depressive symptoms on a continuum. Results could potentially differ with a broader outcome definition capturing subsyndromal depression, and many studies of social capital and depression use scales of symptom severity [5]. However, the studies mentioned above by Ayernor [33] and Ramlagan et al. [32] which used alternative, broader measures of depressive symptoms in the SAGE Ghana and South Africa data still found similar results to those observed in this analysis.

In terms of the positive relationship observed between sociability and depression, depressed individuals are known to become more withdrawn and decrease social interaction [36–38] rather than more social as the current analysis suggests. Yet, given the mixed results linking structural social capital and common mental disorders in the review by De Silva and colleagues [3], the authors posit that they could be influenced by the fact that individuals suffering from mental illness may also be less likely to be actively or regularly working and thus be more available to participate in social activities. These competing forces of withdrawal and availability may therefore muddle the effects. A related explanation could also be that depressed individuals may increase their informal social interactions as a form of overcompensation in an attempt to cope with, distract from, or self-medicate their illness [39]. And perhaps this may also be more relevant in lower income country contexts where professional treatment may not be widely available or may be stigmatized [40]. Conversely, close social contacts of depressed individuals could potentially choose to visit and engage with the afflicted persons more frequently out of concern for their well-being. Evidence for greater involvement and support from social ties in terms of self-rated health has been demonstrated among those in poor health [41].

Additionally, some research suggests that if excessive or within the context of constrained resources, social capital—particularly the bonding variety amongst people of similar backgrounds and statuses—can be burdensome and detrimental for mental health [1, 42]; and Mitchell and LaGory [43] found significantly greater mental distress with increased social participation in the context of a poor, racially segregated southern US community—though they mostly assessed involvement in formal groups. De Silva et al. [39] also observed higher levels of depression and anxiety among individuals with greater community participation and received support in some of the LMICs in their analysis. Thus, it is possible that the degree of sociability may exceed the desired level in older adults in this study—given the LMIC context where there still remains a high degree of poverty and hardship—but may be carried out as a result of personal or cultural obligations and expectations. This reason may be more relevant for Ghana as a lower income country relative to South Africa and could possibly explain the statistical significance observed in the Ghana sample but lack of statistical significance in South Africa although both countries’ estimates for the association between sociability and depression were positive.

The positive relationship between trust and depression in Ghana is also difficult to explain. Though positive correlations between trust and adverse health outcomes are uncommon, one was found with mortality among older Japanese women [44] as well as with depressive symptoms in a longitudinal analysis of South Africans aged 15 years and above [45]. One potential reason could relate to discrepancies between personal feelings of trust and a sense of trust at the contextual level. Low general trust has been observed on the aggregate level among collectivist cultures that emphasize strong in-group ties and have a high degree of familism, or duty and allegiance to kin relations [46]. This leads to a small radius of trust beyond close family ties [46]. As a result, individuals with high trust may be maladapted to a low-trust environment and thus more likely to be depressed. For example, research does suggest that mismatches between personal and societal values can negatively impact mental and physical health [47], and Adjaye-Gbewonyo et al. [45] also observed a cross-level interaction in which the positive association between individual-level trust and depressive symptoms was limited to low trust areas while the association was predicted to be negative in high trust areas. The results of this analysis, however, do not seem to support this explanation of discordance between individual- and contextual-level trust given that South Africans in general had the lowest levels of trust of the 6 SAGE countries (26–32% for men and women), while Ghana ranked in the middle (59–63%) [48]. Another possibility could be a social desirability bias in reporting, namely that depressed individuals in Ghana inaccurately report high degrees of trust.

In terms of the demographic covariates in the adjusted models, the direct relationships between the covariates, age and sex, and depression in the two country samples mirrored the findings in previous analyses [34, 49–51]. In both countries, age also significantly decreased the probability of community engagement and sociability but did not have any significant effects on trust. This conforms with expectations, as physical functioning typically decreases with age in older adults, as does one’s network, and would therefore limit social activity and interaction [52, 53]. Trust, on the other hand, is perception-based and would be unaffected by declining functionality.

In both countries, being female significantly decreased the likelihood of community engagement. However, there was no significant sex difference in informal social participation as represented by the sociability factor, though in Ghana it was trending towards significance (p = 0.094) in the lower direction for females. This is supported by literature which suggests that men are typically more involved in formal social participation [52], although women have been shown to participate more informally and have larger and more close-knit social networks as well as more frequent contact with network ties than men [52–54]. Van Groenou and Deeg [52] suggest that gender differences in resources, such as education, may account for differences in formal and informal participation between men and women. Additionally, lower formal participation in women could potentially be related to cultural norms and gender roles surrounding who may be expected or permitted to participate in community affairs (i.e., meeting with leaders or attending community meetings), and this may also limit women’s ability to engage formally, as well as their greater likelihood to be home-bound due to household duties [55]. Perhaps a bit surprising was that trust was significantly lower in Ghanaian women but not in South Africa. Given greater perceived or actual vulnerability in women compared to men, a finding of lower trust among women is understandable [56]. Nonetheless, lower levels of trust overall in South Africa may make it more difficult to observe significant sex differences, and some studies have demonstrated that women are more trusting than men [57] and others have found no difference [58], which is consistent with the result for South Africa.

### Urban-rural differences in social capital and depression

Results indicated that the measurement of the three social capital dimensions differs between urban and rural residents in both countries, as factor loadings and thresholds for some items had to be allowed to vary between urban and rural groups before measurement invariance could be achieved. Differences in factor loadings and/or thresholds between groups suggest that the interpretation of the dimensions themselves and of the fundamental levels of the items used to measure them are not exactly the same or carry somewhat different meanings between groups. However, all factors did have at least two item loadings and thresholds fixed between the two groups, which would enable drawing legitimate conclusions on group differences in the means of factors [25].

Although there were slight differences in the nature of the relationship between dimensions of social capital and depression between urban and rural residents in the two countries, they were not substantial enough to suggest true effect modification. Thus, the results did not support the hypothesized weaker protective effects of social capital on depression in urban residents and some findings from other studies previously mentioned [12–14]. However, recent studies in China—where depression rates are higher in rural than in urban areas—have conversely found stronger effects of some elements of social capital in urban residents [59, 60].

Despite the lack of meaningful urban-rural differences in the effects of social capital on depression, significant urban-rural differences did emerge in the means of the latent factors. The findings of lower social capital in Ghanaian urban residents could be seen as consistent with assumptions that urban residents are generally more lacking in social connections [61], which is supported by some studies that have demonstrated less social support among urban dwellers [13, 14, 62], a greater likelihood of living alone [63], as well as lower levels of social trust at the ecological level [63, 64]. Nonetheless, empirical evidence of deteriorating social ties in urban settings has also been inconsistent. Some research has shown that the number of ties in urban environments is no different from, if not more than, in rural areas [65]. Levels of participation in organizations and social activities also have not varied significantly across area of residence in some studies [63, 66]. And people in desolate rural areas are also believed to be at risk of social isolation [65]. Some scholars conclude that the nature and composition rather than the amount of social relationships may differ between urban and rural areas, with urban dwellers having more fragmented networks and more social ties and support from friends as opposed to family members, as well as less familiarity with and social support from neighbors [61, 63, 64, 67]. Therefore, findings of no difference or higher average levels of dimensions of social capital in South Africa in this analysis can also be viewed as consistent with this literature. The variations in urban-rural differences in levels of social capital observed between the two countries in this study, as well as in the literature, could also potentially be influenced by the nature and degree of urbanization. South Africa, for instance, is more advanced in its urbanization process than Ghana, with two-thirds of its population currently residing in urban areas but a slower rate of urbanization compared to Ghana, which now has an estimated 56% of its population in urban areas [68].

### Strengths and limitations

A limitation that is important to acknowledge is that the structural model analyzed here is a simplification and does not represent the totality of relationships between dimensions of social capital and depression. For example, reciprocal causation has been documented between social interaction and support and depression [38, 69]. Additionally, many other personal characteristics described above apart from age and sex have an effect on depression and vice versa, and these were also not included in the models. The decision to exclude these additional covariates and bidirectional relationships was made in the interest of reducing complexity in the SEM and facilitating interpretation, as well as ensuring identifiability of the model.

Additional limitations are the limited measures included in the survey for assessing factors such as social support, and, as described earlier, the cross-sectional survey design, which prevents clear ascertainment of the direction of modeled relationships. Furthermore, traditional CFA does not allow for items to load on more than one factor, which is a strong assumption particularly for psychosocial research and may also negatively impact model fit [70]. Nonetheless, models in this analysis generally showed acceptable fit, which provides some degree of confidence in the results and is a strength of the study. And although χ^2^ tests were still significant in all cases, this test statistic is easily affected by sample size and thus cannot be interpreted in isolation given that large samples will tend towards significance [24, 25].

Another strength of the study is the use of a data-driven approach to identify dimensions of social capital through EFA. This allowed for determining population-specific relationships between indicators of social capital rather than applying a standard dimensional structure that may be inappropriate for the data. Furthermore, the use of SEM for data analysis was particularly important. The usual approach to an analysis with multiple indicators of a measure is to sum items to produce overall scores. However, this assumes each item has equal weight, which may not be warranted. It also assumes the item responses can be taken as the true values that are measured exactly without error [71]. SEM has the advantage of simultaneously modeling the relationship between items and their underlying factors as well as the main relationships of interest, thus accounting for measurement error. Moreover, it has an advantage over modeling relationships for each of the items separately in that it reduces the analytic burden if there are numerous items and simplifies the synthesis of results.

## Conclusions

In summary, this analysis provided insight into the structure of social capital and its relationship to depression for urban and rural older adults in understudied African settings. Results of this study suggested that a three-factor solution was favored in the EFA, covering the domains of community engagement, sociability, and trust. Results further demonstrated that the distributions of dimensions of social capital differ between urban and rural residents in Ghana and South Africa even though substantial differences in the magnitude or strength of the association between social capital and depression were not observed. In addition, the relationships between social capital and depression varied depending on the country. Based on the results, it can therefore be assumed that the relationship between social capital and depression is similar within each nation as a whole but not across the two countries, even though the composition of social capital may have some within-country differences depending on urban-rural residence. As suggested previously in the discussion, such cross-country differences are likely related to different conditions within the two countries. Thus, despite the fact that both Ghana and South Africa are middle-income sub-Saharan African nations, differences in a variety of factors such as their physical environments, demographic profiles, cultures, histories, colonial and post-colonial experiences, or economies may have an impact on social capital and how it affects depression.

The implications of the study results are complex, given that the relationships between social capital and depression were not found to be solely beneficial. If these findings hold true, efforts to increase community engagement among older adults, particularly in urban areas, may still have modest positive effects on mental health in both countries; but lower levels of informal social participation among urban compared to rural older adults may actually be protective in terms of depression in Ghana; and easing the burden of informal social activity in South African urban residents in particular may also have positive outcomes for depression. As in other cases in the literature where social capital was found to be positively associated with common mental disorders, these may be economically disadvantaged settings, so simultaneous attention should be given to reducing poverty or alleviating other stresses that cause engagement in social activity to become burdensome for older adults. Alternatively, if depressed older individuals are choosing to engage more informally, this might provide opportunities for trying to capitalize on this activity to maximize the therapeutic potential of such interactions or link afflicted individuals to care. Additionally, promoting trust or identifying and reducing barriers to it may be important in preventing depression in South Africa, with a particular emphasis on trust in rural areas where it is more lacking. This may not be the case, however, for older Ghanaian adults, and determining how to transform trust into an asset would be necessary in this context before attempts at enhancing it can be made.

These findings highlight the variable nature of determinants of health and the need to be cognizant of the language used to describe them, as some factors typically conceptualized as protective may actually confer risk under different circumstances. They also underscore the importance of context and of generating locally relevant evidence in multiple settings to increase the context-specific knowledge base. This will better inform programs and policies that are proposed and implemented to improve health rather than assuming similarity or transferability of evidence from other locations. And in keeping with this observation, comparative research can also enhance our understanding and help to illuminate how and why outcomes differ across settings as well as differences in the effectiveness of programs and policies.

Further analyses are needed to identify which particular elements of the social capital dimensions may be driving the results as well as to establish possible interactions with other factors, such as poverty or wealth and area-level social capital. Future research would also benefit from including additional measures of social capital to determine whether relationships are consistent, and supplementation with qualitative methods could also assist in understanding contextual differences and identifying other factors influencing these findings.
